# Cellulose Nanofibers Extracted From Natural Wood Improve the Postharvest Appearance Quality of Apples

**DOI:** 10.3389/fnut.2022.881783

**Published:** 2022-05-13

**Authors:** Yongxu Wang, Jing Zhang, Xinjie Wang, Tingting Zhang, Fujun Zhang, Shuai Zhang, Yuanyuan Li, Wensheng Gao, Chunxiang You, Xiaofei Wang, Kun Yu

**Affiliations:** ^1^Xinjiang Production and Construction Corps Key Laboratory of Special Fruits and Vegetables Cultivation Physiology and Germplasm Resources Utilization, Department of Horticulture, College of Agriculture, Shihezi University, Shihezi, China; ^2^National Key Laboratory of Crop Biology, MOA Key Laboratory of Horticultural Crop Biology and Germplasm Innovation, College of Horticulture Science and Engineering, Shandong Agricultural University, Tai'an, China; ^3^Shandong Agricultural Technology Extension Center, Jinan, China

**Keywords:** fruit appearance quality, fruit cuticular wax, fruit shelf life, cellulose nanofibers, postharvest preservation technology

## Abstract

To prolong the shelf life of perishable food with a simple and environmentally friendly postharvest preservation technology is one of the global concerns. This study aimed to explore the application value of biological macromolecule natural cellulose nanofibers (CNFs) in extending the postharvest fruit shelf life. In this study, 0.5% (wt%) CNFs were prepared from natural wood and coated on the surface of early-ripening apple fruits. After 10 days of storage at room temperature, the results revealed that the shelf life of apple fruits with CNF coating was significantly prolonged, and the fruit appearance quality improved. The invisible network structure of CNFs in the fruit epidermis was observed under an atomic force microscope (AFM). The gas chromatography and mass spectrometry (GC-MS) analysis showed that CNFs significantly promoted the formation of epidermal wax, especially fatty alcohols, during storage. In addition, the CNFs remarkably promoted the upregulation of genes related to the synthesis of cuticular wax of apple. In conclusion, this study provides an environmentally sustainable nanomaterial for post-harvest preservation of horticultural products, and also provides a new insight into the effect of CNFs on postharvest storage of apple fruits.

## Introduction

Postharvest loss of fruits and vegetables is still a concern at present (1). According to the statistics of Food and Agriculture Organization of the United Nations (FAO), about 40%−50% of fruits and vegetables are wasted after harvest every year, especially in developing countries (2). Fruits and vegetables are prone to decay and spoilage, implying that they do not have a satisfactory shelf life after harvest. Postharvest spoilage is a complex process involving respiration, water loss, microbial growth, and ripeness. The traditional ways to extend the postharvest shelf life of fruits and vegetables include physical preservation (e.g., cold storage, packaging, radiation treatment, and so on) and the use of chemical preservatives (e.g., spraying wax, spraying calcium chloride, using 1-MCP, and so on). However, many countries and regions lack large storage facilities for fruits and vegetables; also, the management mode of transportation exacerbates the loss of food (2). Further, consumers may be wary of treating fruits and vegetables with chemical preservatives because of unexpected health hazards (3). Therefore, a healthy and environmentally sustainable biomass method to extend the shelf life of fruits and vegetables needs to be developed.

Nanomaterial technology has made great strides in recent years by controlling the properties of materials at the nanometer scale, thus achieving high space utilization and microenvironment management (4, 5). Therefore, nanotechnology seems promising to extend the shelf life of fruits and vegetables (6–8). Cellulose is the most widely distributed and abundant organic macromolecule in nature. Cellulose with a nanometer structure is defined as nanocellulose (9). Cellulose nanofibers (CNFs) are a kind of nanocellulose, 5–7 nm in diameter, with a length of hundreds of nanometers, high aspect ratio, and characteristics of a strong hydrogen bonding network (10). The Joint Expert Committee on Food Additives of FAO/WHO (JECFA) has listed rod type microcrystalline cellulose (INS No. 460i) as a food additive (11). For the long fiber type (i.e. CNF), although it has not been listed in with INS number, however, some official patent documents mention the application of these CNF in food industry (12, 13). They are often used as dietary fiber food stabilizers in recent years (14–16) because of their good gas barrier property (17) and a moderate amount of water vapor permeability (18).

In recent years, the application of nanocellulose in postharvest preservation of horticultural products has been widely concerned. For example, CNF-based emulsion coating can effectively prolong postharvest storage time of bananas (19); The cellulose nanocrystals (CNC) could enhance pear postharvest storage performance at room temperature and cold storage by enhancing chitosan coating (20); Kwak et al. reported that a cationic salt stabilized carboxymethylated cellulose nanofibers (CM-CNFs) edible film could protect strawberries from microbial contamination and extend shelf life (21). Pacaphol et al. reported that a single CNF coating could improve fresh-cut spinach (*Spinacia oleracea L*.) storage by inhibiting the respiration rate (22). In addition, CNFs have strong adhesion and good waterproof performance, which can protect the cuticle of cherry fruit sand reduce cherry rain cracking (23). However, these researches mainly focused on the application level of CNFs, but few explore the physiological and biochemical mechanism of fruits and vegetables after harvest.

In this research, early-ripening apples was selected as experimental materials, these apples have a very short ripening period, but most early-ripening cultivars have a short shelf life and can only be sold fresh rather than stored for a long time (24), prepared CNF colloidal suspension from natural wood, and tried to spray CNF colloidal suspension on the surface of apples. Since CNF first touches the wax layer of fruits after spraying, it is necessary to study the chemical composition of wax in fruits during storage. The cuticular waxes of fruits are the first barrier against biological and abiotic stress (25), and waxes are closely related to postharvest quality of fruits (26, 27). The aim of this study was to investigate the effect of CNF coating on the changes of cuticular wax composition and physiological changes of perishable fruits during the shelf life stored at room temperature. This study provided a new idea for the application of nanocellulose in postharvest fruit preservation and also provided some insights into the effect of nanocellulose materials on the chemical composition of wax in fruit epidermis.

## Materials and Methods

### Fruit Materials

Mature apple fruit (*Malus domestica*, ‘Liao Fu’) were harvested from a local apple orchard in Tai'an, Shandong Province of China on June 20, 2021. The tree was 8 years old, the conventional management, the growth results were good. The standards for picking the samples were: 1, apple skin has no mechanical damage, 2, no physiological diseases, and 3, fruit size and appearance are uniform. The apples were picked into polyvinyl chloride bags after harvest. On the day of picking, the apples were sent to the State Key Laboratory of Shandong Agricultural.

### CNF Preservative Agent Preparation and Apple Fruit Treatment

The CNF were prepared from waste wood of apple orchard (same origin as apple fruits). The wood was firstly chopped into some small chips, and then these were bleached with 1% (w/v) sodium hydroxide (NaOH) solution for 4 h at 85°C (28, 29). After that, the obtained cellulose was washed with acid and distilled water until the solution was neutral and dried in a vacuum oven at 60°C for 12 h. The obtained dry cellulose was added into distilled water (mass fraction 1% w/v) and stirred in a high-speed blender (Y25, YULDOR, Germany) at 25,000 rpm for 30 min to disperse it in water. Then it was homogenized by a high-pressure homogenizer (ATS-AH2010, ATS Engineering Inc., Canada) at pressure levels ranging from 40 to 140 MPa and for up to 50 HPH cycles. The homogenized solution was quickly frozen and transferred to vacuum freeze dryer (FDU-1110, EYELA, Tokyo) for 48 h to obtain CNF powder.

All fruits were randomly divided into two groups of one hundred each. Weigh 0.5 g of CNF powder and add it into 100 mL distilled water, and then conduct ice bath ultrasonic treatment (800 W, 30 min) to prepare CNF colloidal solution. The CNF colloidal solution (0.5% w/v) was sprayed on the surface of the postharvest apple fruits and then stored at room temperature (± 25°C) with 45–75% relative humidity for 10 days. The control fruits were sprayed with the same volume of the distilled water and the similar storage condition for 10 days.

### Fruit Glossiness and Color Measurement

Micro-TRI-gloss tester (BYK-4563, Germany) wax used to determine fruit surface gloss. The measuring mouth of the instrument was aligned with the equatorial plane of the fruit for testing, and 10 points were randomly selected for each apple for testing. The color of the apple surface was determined by a chroma meter CR-400 (Konica Minolta Sensing Inc., Japan). Lightness (L^*^), Redness (a^*^) and Yellowness (b^*^) values were recorded on the equatorial surface of the fruit.

### Assessment of Fruit Quality Related Indexes

For the determination of fruit quality indicators, each treatment group carried out six biological experiments. An experiment was performed every 2 days.

#### Firmness

The fruit firmness was measured by fruit texture analyzer (Stable Micro Systems, Godalming, UK) with a P/2 columnar probe (diameter: 2 mm) was used to measure fruit firmness. The pretest velocity was 2 mm s^−1^, measurement velocity was 1 mm s^−1^, and post-test velocity was 5 mm s^−1^. The data were automatically analyzed and calculated by Texture Exponent 32 software. Firmness readings are expressed in Newtons (N), the average of the measured values recorded at four locations in the equatorial plane of each fruit (30).

#### Weight Loss

The weight loss of fruit during storage was calculated by weighing method (BH-300, Excell Inc., Shanghai, China). Weight loss was calculated as: W_0_-W_1_ / W_0_ × 100%, where W_0_ is the initial weight, and W1 is the final weight. Weight loss was expressed as a percentage (%) of fresh weight (30).

#### Sugar-Acid Ratio

Apples were randomly selected from each group, and total soluble solids (TSS) and titratable acids (TA) were determined from each apple. TSS was determined with a digital hand-held refractometer (Atago PAL-1, Japan). The value of TA was determined by acid-base titration and titrated with 0.1 m NaOH to pH 8.1. The result was expressed as a percentage of malic acid. The sugar-acid ratio is calculated as the ratio of TSS to TA.

#### Pericarp Relative Cell Membrane Permeability

The pericarp relative cell membrane permeability was carried out according to the method described in previous studies with minor modifications (31, 32). Pericarp tissue of the same thickness (about 1 mm) was peeled and an 8 mm round punch was used to cut the pericarp tissue. Ten pieces of tissue were taken as a group, and the thickness of the peel was consistent and damage was minimized. This process was repeated 10 times for each period of fruit sampled. The pericarp tissue was transferred into a 50 ml conical flask and 30 ml distilled water was added, followed by vacuum extraction for 30 min. S1 conductivity (DDS-307A, INESA Scientific Inc., China) was measured after the pericarp sank to the bottom. Next, pericarp tissue samples were boiled for 20 min. S2 conductivity was measured after cooling the tissue to room temperature (± 25 °C). Relative conductivity (S) was calculated: S = S1/S2 × 100%.

#### Respiration Rate

The respiration rate of fruit was measured by CO_2_ detector (Testo 535-CO_2_, Testo Inc., Shanghai, China). Three apple fruit samples randomly selected were placed in a 2 L glass jar sealed with sellotape for 2 h at room temperature (± 25 °C). Then, the CO_2_ sensor was inserted into the glass jar to measure respiration (33). The respiration rate of the fruit was denoted as μL g^−1^ h^−1^. Six biological replicates in each group.

#### Ethylene Production

Fruit were taken from each treatment and assessed for ethylene production. Weigh the apples in each group, then, three fruit of each treatment randomly selected were placed individually into 2 L glass jars (Seal with sellotape) for 6 h, 25 °C. One mL gas sample was taken from the head space using a gastight syringe and injected into a gas chromatograph (GC-2014, Shimadzu, Japan), equipped with a Shimadzu GC-2014 flame ionization detector (GC-FID) and GDX-502 column. The temperatures of chromatograph column, injector and GC-FID were 70 °C, 140 °C and 200 °C, respectively. The carrier gas N_2_ flow rate was 30 mL min^−1^, the H_2_ flow rate was 30 mL min^−1^, and the air flow rate was 300 mL min^−1^. The ethylene production rate of the fruit was calculated (μL g^−1^ h^−1^): (*c* × V)/(m × t × 1000), wheres *c* is the amount of ethylene in the samples (μL L^−1^); V is the volume of the glass container (mL); t is measuring time; m is the sample weight. Six biological replicates in each group.

### Electron Microscopic Observations

#### Scanning Electron Microscopy (SEM)

The microstructures of cuticular wax in apple were observed by SEM followed by Yang et al. (34). A sharp blade was used to cut about 3 cm^2^ of pericarp tissue. Subsequently, the pericarp samples were then frozen using liquid nitrogen, and then transferred to FDU-1110 vacuum freeze dryer (EYELA, Tokyo, Japan) for 24 h for tissue dehydration. The freeze-dried samples were coated with platinum target JFC-1600 ion sputtering apparatus (JEOL, Tokyo, Japan). Use JSM-6610 SEM (JEOL, Tokyo, Japan) to observe the epidermal wax structures.

#### Transmission Electron Microscopy (TEM)

CNF suspension with uniform ultrasonic treatment (0.01%, w/v) for 30 min was dropped onto the carbon support film (230 mesh) and dried at room temperature. TEM images were carried out on microscope (Tecnai G2 F20, FEI Inc., USA) at 200 kV.

#### Atomic Force Microscopy (AFM)

CNF suspension with uniform ultrasonic treatment (0.1%, w/v) for 30 min was sprayed on mica sheets of about 1 cm^2^. After drying with water, AFM microscope (Bruker Dimension Fast Scan) was used for observation. The AFM measurements were performed in tapping mode.

### Cuticular Wax Extraction and GC–MS Analysis

The wax extraction experiment was slightly modified according to Yang et al.'s method (34). The apples were soaked in 200 mL chloroform for 45 s, then the extracts were rotated and evaporated into a glass bottle. The apples were then dried under nitrogen flow. The total of apple cuticular wax amounts was determined calculated using the following formula: Wax amounts (μg cm^−2^) = (W_1_-W_0_)/S, where S is total surface area of 5 apples; W_1_ is weight of vials and wax; W_0_ is initial weight of the vials.

The wax was re-dissolved in 10 mL of chloroform: methanol (10:1, v/v) with internal standards of n-Tetracosane (1 mg mL^−1^, Sigma-Aldrich, St. Louis, MO, USA). One milliliter of sample was dried under a stream of nitrogen and then derived with 300 μL of bis-N, N-(trimethylsilyl) trifluoroacetamide (BSTFA; Sigma-Aldrich, St. Louis, MO, USA) for 40 min at 70°C. After removing the BSTFA under a stream of nitrogen, the derivatives were dissolved in 1 mL chloroform for the GC-MS analysis. The quantitative and qualitative analyses were carried out by GC-MS (QP-2010 plus, Shimadzu, Tokyo, Japan) under the same conditions as GC except that helium was used as the carrier gas. Wax compounds were identified by matching their electron ionization mass spectra (70 eV, m/z 50–850) with those from the NIST17 MS library. A capillary column (DB-5MS, 30 m, 0.25 mm i.d., 0.25 μm film) was used to separate the compounds using N_2_ as the carrier gas. The column temperature was programmed with an initial temperature of 120°C for 2 min. The temperature was increased by 10°C min^−1^ to 190°C, increased by 2°C min^−1^ to 216°C, held for 5 min at 216°C, increased by 3°C min^−1^ to 300°C, and finally held for 5 min at 300°C. The detector gases were hydrogen and air at flow rates of 50 ml and 400 mL min^−1^, respectively. The flame ionization detector (FID) temperature was 320°C. Quantification was based on the FID peak areas and the internal standards.

### Real-Time Quantitative RT-PCR (qRT-PCR)

The sample preparation procedure for qRT-PCR was slightly modified according to the method of An et al. (35). The RNA was extracted from pericarp tissue according to the method provided by the RNA plant plus reagent kit (Tiangen, Beijing, China). Then, the single-stranded DNA was synthesized by reverse transcription using a reverse transcription kit (Takara, Dalian, China). The single-stranded DNA concentration was diluted to 1.8 ~ 2.4 ng μL^−1^. The diluted cDNA was used to examine the qRT-PCR and *MdActin* served as an internal control. The relative expression of the target gene was calculated by 2^−ΔΔCt^. The qRT-PCR assays were conducted with the Ultra SYBR mixture (SYBR Green I, Takara, Japan) using an ABI7500 qRT-PCR system. The primers used in this study were synthesized by Sangon Biotech (Supplementary Table S1).

### Data Analysis

All statistical analyses were carried out using the software OriginPro 2017. Two tails student's *t*-test and Duncan's new multiple range test were used to analyze the significant differences between different treatments and different storage periods.

## Results and Discussion

### Schematic Diagram of CNF Preparation and Effect of CNF Antistaling Agent on Apple Fruits

**Figure 1:**
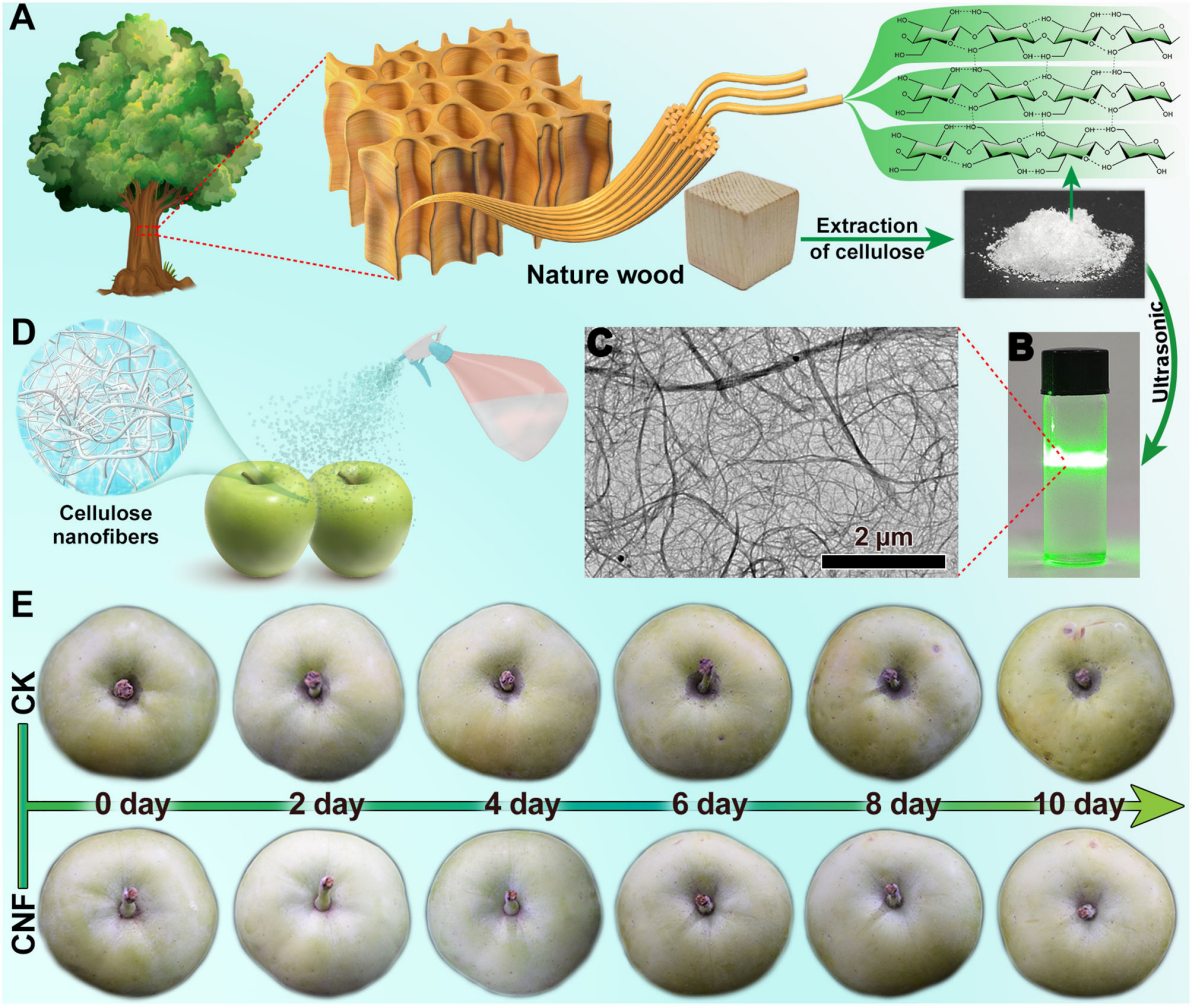
**(A)** Schematic of cellulose preparation from natural wood. **(B)** Cellulose nanofiber colloidal solution. **(C)** Transmission electron microscope (TEM) image of CNF. **(D)** Schematic of CNF preservatives agent spraying on fruits. **(E)** Effect of CNF preservatives agent on apple fruits.

Figure 1A graphically elucidates the cellulose derived from the natural wood. Cellulose was a green renewable material, and the nanoscale cellulose was highly promising in healthy and safe food (14). Subsequently, the CNF colloid solution (Figure 1B) was prepared by ultrasonication, which displayed the visible Tyndall effect. The transmission electron microscope (TEM) image of the colloid solution (Figure 1C) exhibited that the nanofiber networks were composed of interlaced CNFs of diverse size. The nanocellulose colloid solution was used as the preservative for apple fruits. The simple spraying treatment could make the CNF coat on the surface of the fruits (Figure 1D). The specimen treated with the CNF colloid solution was labeled as CNF. No visual change was observed in the appearance of fruits treated with CNF preservative, which benefited from the high transparency of nanoscale cellulose. In a control experiment, equal amounts of water were sprayed on the samples (named as control) in the same way. The fresh-keeping effect of the CNF antistaling agent on the fruits was evaluated by an experiment with different storage times using the climacteric apple fruits. Figure 1E displays the changes in the appearance of the fruits recorded by taking a picture every 2 days. In the initial stage of storage (0 day), the control and CNF specimens presented a similar appearance of fruits, which also reflected the invisibility of the CNF fresh-keeping agent. After 10 days, the control and CNF specimens presented a distinct appearance. The surface of the control group apple fruit revealed plentiful dimples and its skin color was obviously darker with some enzymatic browning spots, indicating decay. In contrast, the apple fruits treated with the CNF preservative showed an appearance similar to that in the initial stage.

The results are similar to what has been observed in strawberries (21), spinach (22) and fresh-cut apples (36). The CNF has good wettability (18) due to the presence of a large number of hydroxyl groups (-OH) on the surface of the material, which form stable hydrogen bonds with the H_2_O molecule. This strong network of hydrogen bonds can lock up the surface environment of the fruits surface and the moisture released by the epidermis pores. The results revealed that the CNF preservative possessed good freshness retention.

### Effects of CNF Preservatives on Glossiness and Color of Fruits During Shelf-Life

**Figure 2:**
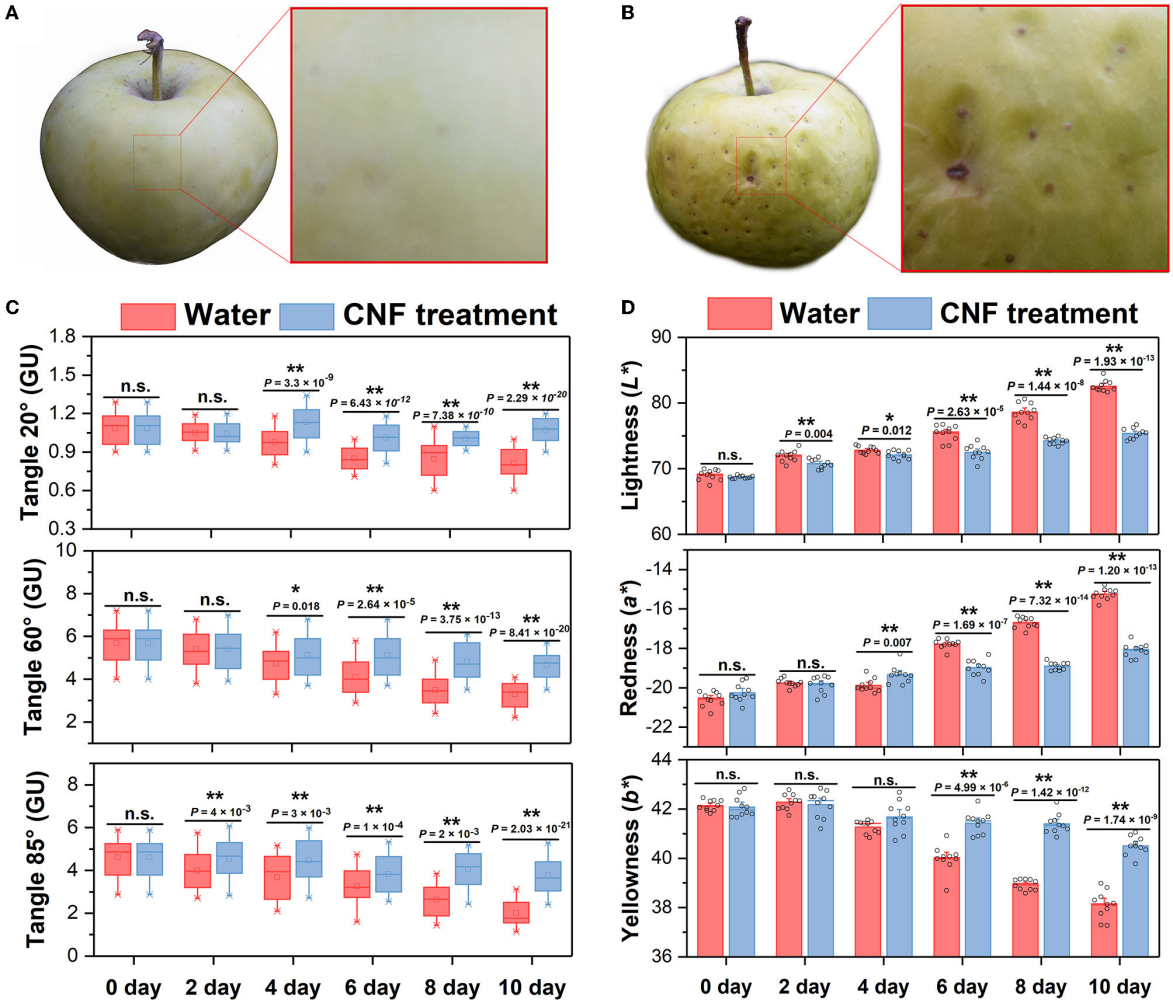
**(A)** Appearance of CNF-treated apple fruits after 10 days. **(B)** Appearance of the control group apple fruits after 10 days. **(C)** Epidermal glossiness of apple fruits. Box edges represent the 0.25 and 0.75 quantiles, and the bold lines indicate median values. Whiskers indicate 1.5 times the interquartile range. The asterisks indicate a statistically significant difference (two-tailed Student *t*-test, **P < 0.01). n.s. represent no significant difference (*P* > 0.05). Ten biological replicates. **(D)** Fruit skin color. The asterisks indicate a statistically significant difference (two-tailed Student *t* test, *P < 0.05, **P < 0.01). n.s. represent no significant difference (*P* > 0.05). Error bars, mean ± standard deviation. Ten biological replicates.

Generally, the evaluation of fruit freshness includes several indicators such as appearance color, fruit firmness, weight loss, total soluble solids and total titratable acid. The freshness degree of the fruits was intuitively reflected by their skin phenotypes in which the epidermal glossiness and color were closely related to the appearance acceptability (24, 37). Figures 2A,B compares the phenotypes of the fruit epidermis after 10 days. The apple fruits treated with CNF preservatives had much lighter spring-green skin than those in the control group with a flat surface, which reflected good freshness and quality. In contrast, the skin of the control apple fruits was pale yellow and wrinkled, indicating its inferior fruit quality. Apple epidermis gloss is one of the most important appearance qualities of apple. During postharvest storage, apple cuticular wax components will change due to internal physiological changes, resulting in greasiness and other physiological diseases that affect fruit commercial value (34, 38, 39). Figure 2C shows the epidermal glossiness of these apple fruits at different preservative times and three angles of 20°, 60°, and 85°. As the retention times increased, the skin glossiness of apple fruits treated with CNFs presented a relatively stable trend for the three angles, continuing with the impression of good fruit quality. In contrast, the epidermal glossiness of 20° and 60° angle of apple fruits in the control group was significantly lower (*P* < 0.05) than that of CNF treatment on the 4th day of preservation; the sharpest drop was recorded at 85° angle (this was near the normal-viewing angle of the human eye). This made the poor fruit quality easier to note. Moreover, the results agreed with the phenotype observation (Figure 1E). When the retention time increased to the 10th day, compared with that in the CNF treatment group, the fruit glossiness at 20°, 60° and 85° angle in the control group decreased by 24.2%, 30.0% and 47.1%, respectively, corresponding to the inferior appearance of the fruit (Figure 2B). The result indicated the excellent preservative effect of CNFs.

During postharvest storage, fruits and vegetables will change color due to internal physiological changes (40). The epidermal color at different retention times is shown in Figure 2D. The recorded color parameters were lightness (L^*^), redness (a^*^), and yellowness (b^*^). During storage, compared with CNF treated fruits, L^*^ and a^*^ values were significantly increased (*P* < 0.05) on the 2nd and 4th day, respectively; while b^*^ values were significantly lower (*P* < 0.05) than those in the CNF-treated on the 6th day of storage. A comparison with the control group revealed that the apple fruits treated with CNFs exhibited a less pronounced change in epidermis color parameters, which was consistent with the observed appearance (Figure 1E). The color parameters and glossiness measurements indicated that CNFs could help preserve apple fruit appearance quality.

### Microstructure and Coating State of the CNF Preservative Were Analyzed Using an Electron Microscope

**Figure 3:**
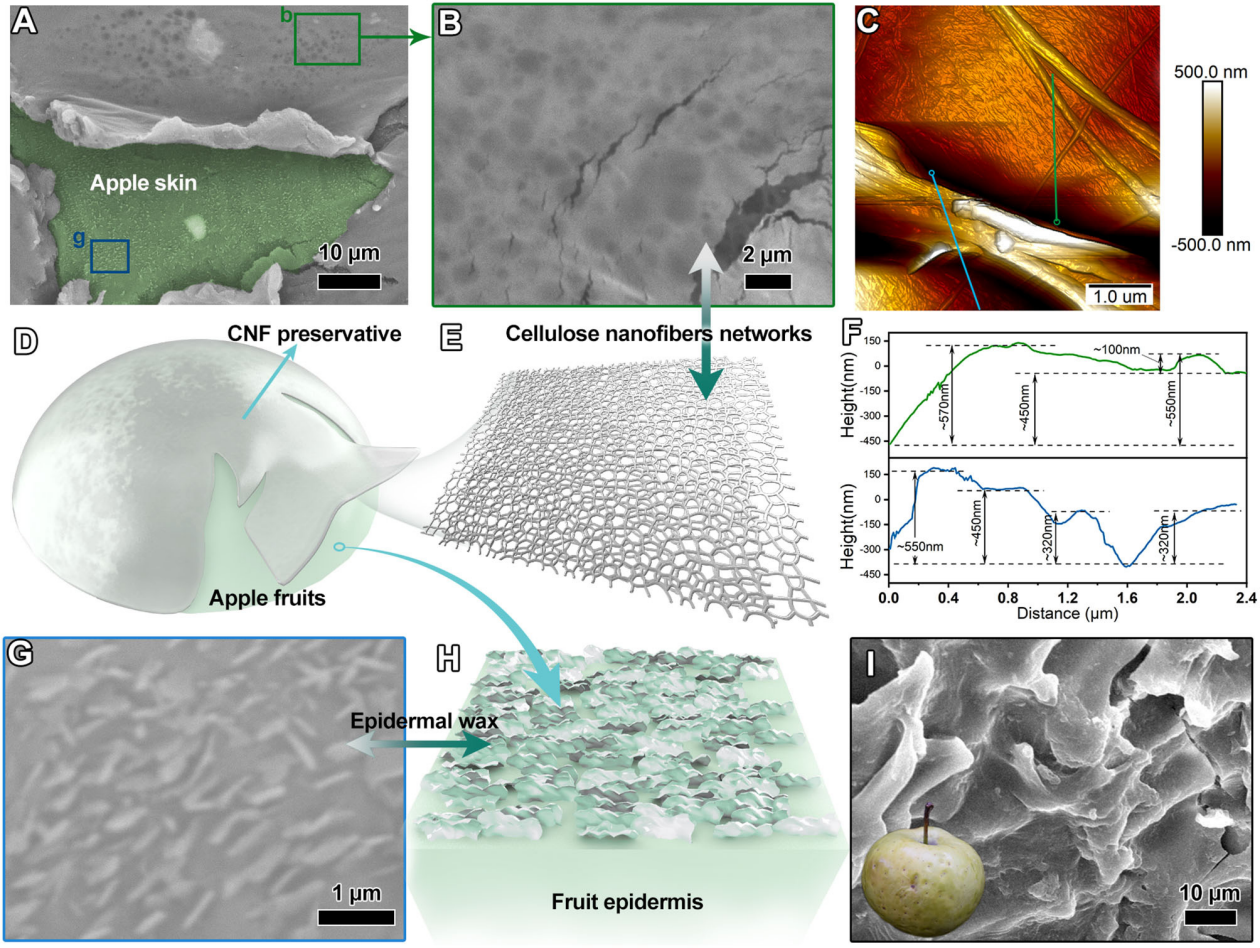
**(A,B)** SEM images of the epidermis of apple fruits treated with CNF. **(C)** Atomic force microscope (AFM) image of the CNF preservative. **(D)** Diagram of apple fruits with the CNF fresh-keeping agent. **(E)** Microstructural schematic of the CNF preservative. **(F)** Height profiles (corresponding to indication lines in AFM image). **(G)** SEM image of the CNF-treated apple epidermis. **(H)** Corresponding schematic diagram of the fruit epidermis. **(I)** SEM image of the control apple epidermis (inset is apple fruit picture after retention of 10 days).

The epidermal SEM image of the apple fruits treated with CNF preservatives (Figure 3A) showed that the cellulosic lamellar structure covered the surface of apple fruits skin (the region highlighted by light green color). The apple fruit epidermis presented a relatively flat formation. Further examining the cellulosic lamellar structure located on the surface of apple fruit skin (Figure 3B) revealed that it was composed of dense cellulose networks. The network constructed by interconnected nanofibrils was similar to spider webs, graphically shown in Figure 3E. Meanwhile, the cellulose preservative layer on the surface of apple fruits was measured using the atomic force microscope. Figure 3C shows the pyknotic CNF conglomeration with the thickness of several hundred nanometers. The corresponding height profiles revealed that these CNF diameters were mainly <100 nm (Supplementary Figure S1), and the cellulose laminate architectures had varying thicknesses of 100–600 nm (Figure 3F). The lamellar structure stacked by CNF networks enabled the surface of apple fruits to form a protective layer similar to that covering a nanoscale invisible armor (illustrated graphically by Figure 3D). The epidermis morphology of apple fruits treated with CNF preservatives is shown in Figure 3G. The numerous squamous sheets with a relatively aligned arrangement were detected on the surface of apple fruit skin, which belonged to the epidermal wax (Figure 3H). The cuticular wax of fruits is a key factor for the fruit gloss (34, 41). The cuticular wax was significant to the fruit quality. The morphological observation revealed good construction of the cuticular wax in the apple fruit treated with CNF preservatives, which suggested good quality. In contrast, the epidermal morphology of the fruit samples treated only with water was bumpiness with apparent splits (Figure 3I). In addition, Supplementary Figure S2 further shows the difference in the epidermal wax morphology after 10 days of CNF treatment compared with that in the control group. We observed that the epidermal wax crystal particles of CNF-treated fruits (Supplementary Figure S2B) were larger than those in the control group (Supplementary Figure S2A). The results revealed that the CNF preservative layer could protect the cuticular wax microstructure and maintain the freshness of apple fruits.

### Effect of CNF Preservative on Wax Content of Fruit Epidermis

**Figure 4:**
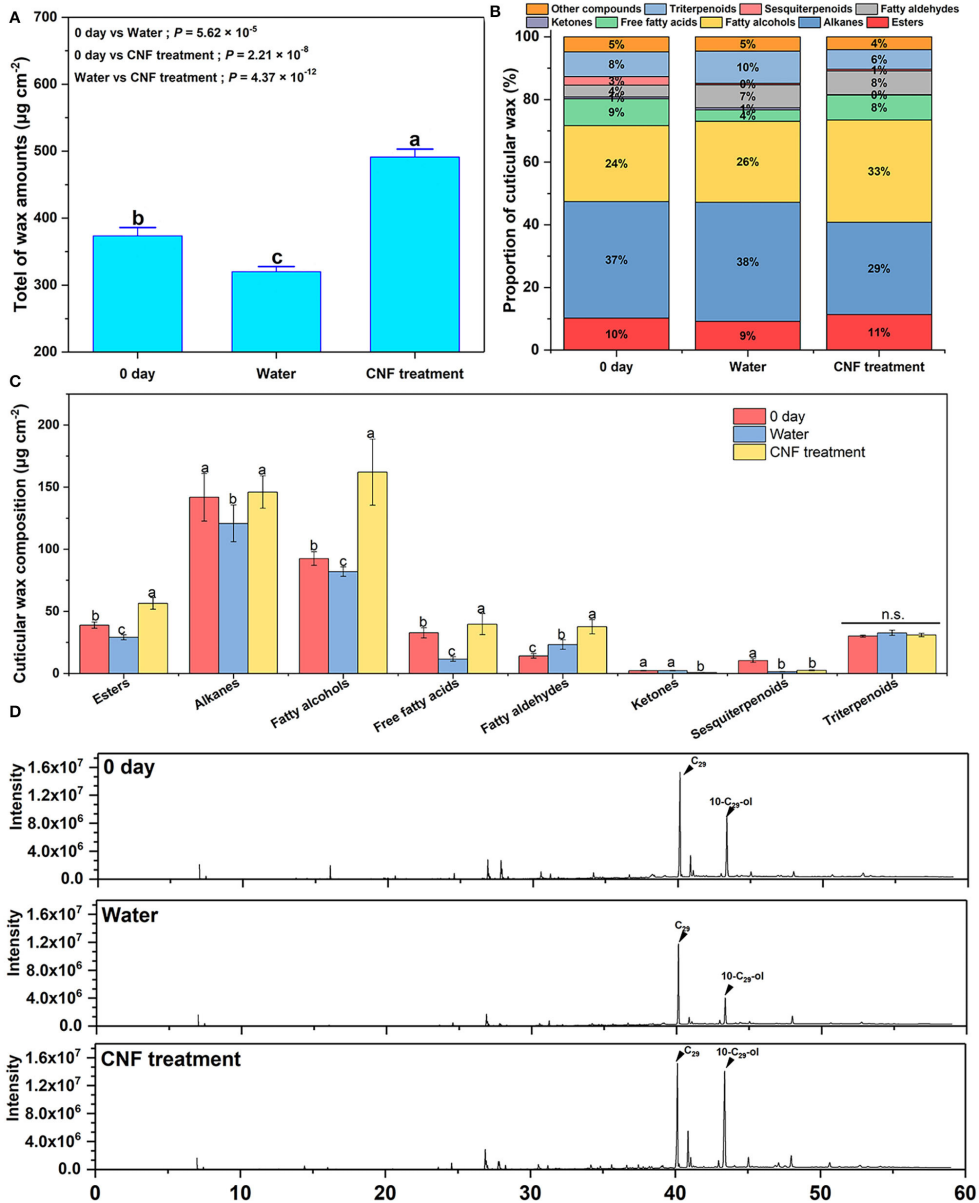
**(A)** Total wax amounts. Different letters represent significant differences according to Duncan's new multiple range test (*P* < 0.05). Error bars, mean ± SD, six biological replicates. **(B)** Relative contents of epidermal wax components in fruits after harvest and after 10 days of different treatments. **(C)** Content of each component in the cuticular wax. Different letters represent significant differences according to Duncan's new multiple range test (*P* < 0.05). n.s. represents no significant difference (*P* > 0.05). Error bars, mean ± standard deviation, six biological replicates. **(D)** Main cuticular wax compounds detected by GC–MS.

Cuticular wax plays an important role in maintaining the postharvest storage quality of horticultural crops (27, 42, 43). The cuticular wax of apples is generally divided into two layers, which are composed of aliphatic compounds and terpenoids (38, 41, 44). The apple epidermal wax was extracted 0 day after harvest and 10 days after storage, and qualitative and quantitative detection was performed using the GC–MS system to further understand the effect of CNF preservatives on the epidermal wax. Figure 4A depicts the total wax content of the 0-day samples, the control samples, and CNF-treated fruits. The mass of postharvest apple fruits decreased during storage; the cuticular wax weight loss was related to the phenomenon (27, 44). The waxes in the cuticle of plant organs play an important role in limiting non-stomatal water loss (45). Among them, intra-cuticle waxes, which are mainly composed of ultra-long chain fatty acids and their derivatives, play the role of transpiration barrier (46). Indeed, the total wax of the control group fruits after 10 days of storage was evidently less than the pristine fruits. Intriguingly, the wax content of samples treated with CNFs was even more than that of the original fruits (0 day). The increase in the total wax content commonly occurred in the phenological stage (42). Therefore, the results suggested that the apple fruits treated with a CNF preservative agent could even increase the bioactivity of epidermal cells during storage.

To understand the changes in wax composition of fruit epidermis, the GC-MS was used to detect the fruits at the initially stages and the end of stage. A total of 41 compounds, including alkanes, fatty alcohols, fatty aldehydes, free fatty acids, esters, ketones, sesquiterpenoids, and triterpenes, were identified and quantified throughout the storage period (Supplementary Figure S3, Supplementary Table S2). Figure 4B shows the cuticular wax major components and their percentage change in pristine samples and after 10-day storage. The essential chemical components of epidermal wax in all samples were esters, alkanes, fatty alcohols, free fatty acids, ketones, fatty aldehydes, sesquiterpenoids, triterpenoids, and other compounds. In the control samples, the free fatty acid proportion evidently decreased and the fatty aldehydes obviously increased because of response to the water loss stress of the fruits. Compared with the apple fruits treated with water, the samples treated with CNF preservatives presented a different proportion of wax chemical components, in which the percentages of fatty alcohols and free fatty acids were distinctly higher. Furthermore, the wax content per unit area is shown in Figure 4C. Compared with the fruits before storage (0 day), only the wax content per unit area of fatty aldehydes obviously increased in the control samples after 10 days, which corresponded to the drastic increase in their proportion. However, except for ketones and sesquiterpenoids, the contents of other waxes per unit area in the CNF-treated apple fruits were higher than those in the fruits after 0-day storage. Among these, the fatty alcohol content increased the most, especially 10-nonacosanol (10-C_29_-ol) (Figure 4D). The contents of fruits treated with CNFs were 59.92 μg cm^−2^ and 48.31 μg cm^−2^ higher than those in the control fruits and those after 0-day storage (Supplementary Figure S3B, Supplementary Table S2). The fatty alcohols could efficiently facilitate the generation of epicuticular wax crystals (26). Thus, the skin of the CNF-treated apple fruits had plentiful wax crystals, as observed in Figure 3G. Meanwhile, the contents of alkanes and fatty alcohols per unit area of CNF-treated fruits were higher than those in the control samples, benefiting the impermeability of the treated fruit cuticles (47). These results indicated that the CNF-treated apple fruits possessed the better water-retaining epidermis with good freshness.

### Effect of CNF Preservative on Fruit Quality During Shelf-Life

**Figure 5:**
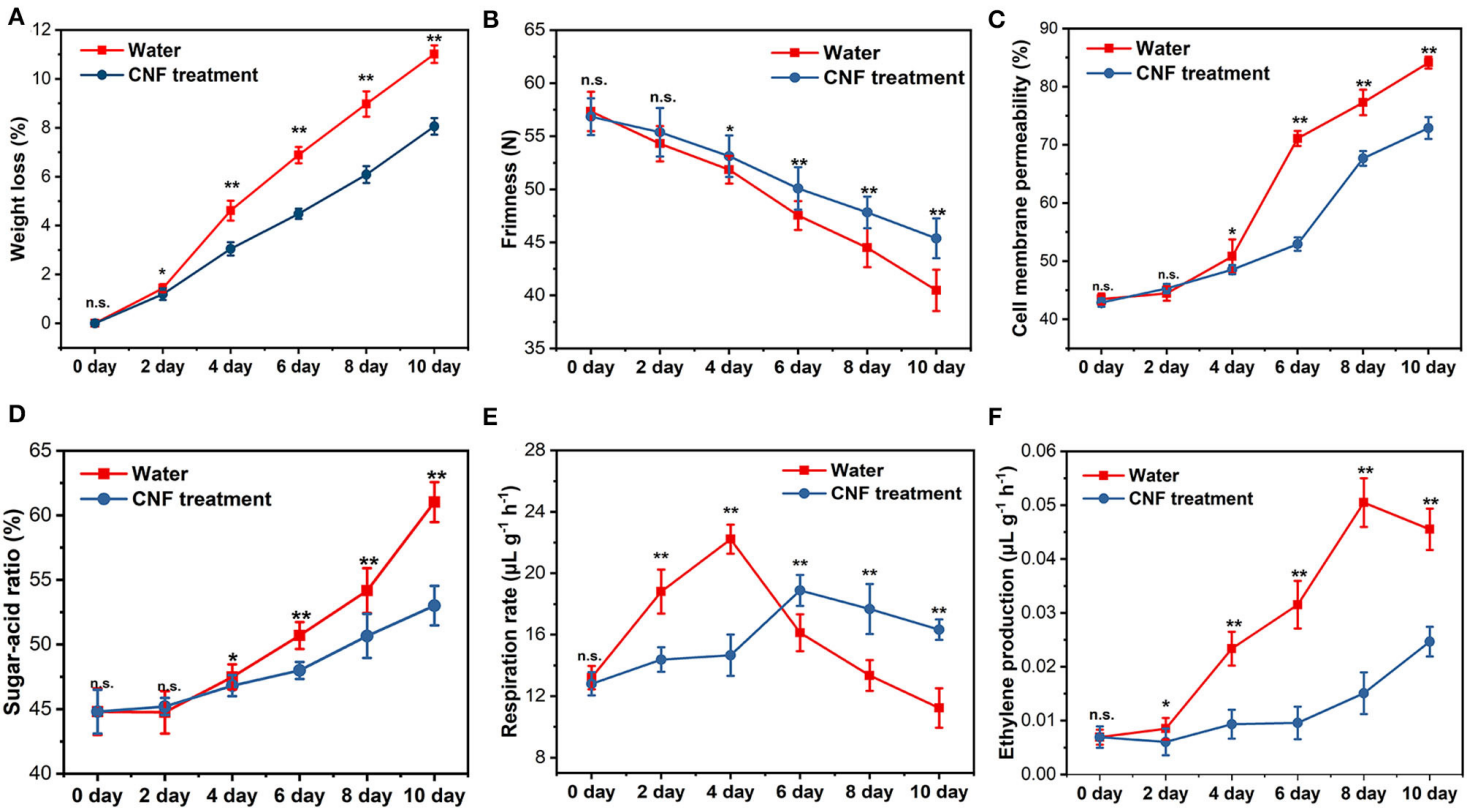
**(A)** Weight loss, **(B)** firmness, **(C)** cell membrane permeability, and **(D)** sugar–acid ratio. **(E)** Respiration rate and **(F)** ethylene production. The asterisks indicate a statistically significant difference (two-tailed Student *t* test, *P < 0.05, **P < 0.01). n.s. represent no significant difference (*P* > 0.05). Error bars, mean ± standard deviation. Six biological replicates.

Fruits and vegetables are semi-living tissue after harvest, so they continue their biological processes such as dormancy, transpiration and respiration (48). Besides the properties of the apple epidermis, the comprehensive characteristics of the whole fruit (including weight loss, firmness, respiration rate, and so on) were further analyzed (Figure 5). Figure 5A shows the changes in the weight loss of these whole apple fruits with storage time. The fruits lost water after harvest, which greatly affected their economic and sensory value. Compared with the control fruits, the apple fruits with CNF coating had a significantly lower weight loss (*P* < 0.05) on the 2nd day of storage. At the end of storage, the water loss rate of CNF-treated fruits was 2.95% lower than that of the control, implying that the CNF preservative helped retain the water that was essential to the freshness of the fruit. The fruit firmness was an important trait that affected consumer acceptance, fruit transportability, and shelf-life. Figure 5B reveals that the firmness of the fruits treated with CNFs was markedly higher (*P* < 0.05) than that of the control fruits after storage for 4 days. The firmness in control decreased within a range of 40.47 N ± 1.94 N after 10 days of storage. Meanwhile firmness in fruit treated with CNF remained relatively steady with a value of 45.37 N ± 1.88 N after 10 days of storage, indicating that the CNF coating contributed to delaying softening and maintaining the firmness of fruits. The water content and fruit firmness were closely associated with the constituents and properties of the cell wall. As shown in Figure 5C, the cell membrane permeability increased continuously in different treatment groups during fruit storage. After 4 days of storage, the cell membrane permeability of fruits epidermis in control group was 50.81% ± 2.91%, which was significantly higher (*P* < 0.05) than that in CNF group (48.53% ± 0.77%). The results showed that the CNF coating has good water retention and freshness preservation function, coinciding with the results of weight loss of the fruits (Figure 5A). Another key parameter was the sugar-acid ratio, which was commonly used to evaluate the fruit quality. After 4 days of storage, the sugar-acid ratio of the fruits in the two groups was obviously different (Figure 5D). At the end of storage, the sugar-acid ratio of 61.02% ± 1.55% in the control group was distinctly higher (*P* < 0.01) than that in the CNF-coated (53.01% ± 1.52%). The TSS values in the two groups were similar and remained relatively stable (*P* > 0.05) during the storage period (Supplementary Figure S4A). The TA values decreased with the increase of storage time (Supplementary Figure S4B). However, the CNF-treated fruits presented relatively stable TA values and hence a lower sugar-acid ratio compared with the control samples. The result showed that the CNF preservative could efficiently inhibit the generation of acid compounds to maintain the fresh taste of the fruits during storage.

Respiration, as a metabolic process, occurred throughout the development and maturation of fruits, and its existence could engender a postharvest quality loss of the fruits. Figure 5E depicts the respiration rate tendency in the two groups of samples during 10 days of storage. The respiration rate of all samples increased dramatically in the early stages of storage and subsequently decreased rapidly due to the stress of water loss, which was similar to previous findings (21, 29, 49, 50). The difference was that the peak of fruits respiration rate appeared on the 4th day of storage in the control group (22.22 ± 0.948μL g^−1^ h^−1^), while that in the CNF-coated (14.66 ± 1.344μL g^−1^ h^−1^) appeared on the 6th day. Moreover, at the end of storage, the respiration rate of CNF-coated fruits was significantly higher (*P* < 0.01) than that of the control group. The result indicated that CNF preservative could suppress the respiration metabolic process to maintain the freshness of the fruits. The ethylene production was closely associated with the metabolism in fruits, and excess ethylene release rendered rapid firmness loss and physiological disorders (51). Ethylene can change the content and composition of wax in fruit epidermis and thus affect fruit quality after harvest (52). Figure 5F reveals that ethylene production in the CNF-treated fruits was significantly lower (*P* < 0.05) than that in the control group on the 2nd day of storage. During subsequent storage, ethylene release increased sharply in the control group, but relatively slowly in the CNF-coated fruits. These results elucidated that CNF preservative achieved a good fresh-keeping effect by restricting physiological and biochemical processes.

### Changes in the Expression of the Key Genes Involved in Wax Formation and Response to CNF Preservative

**Figure 6:**
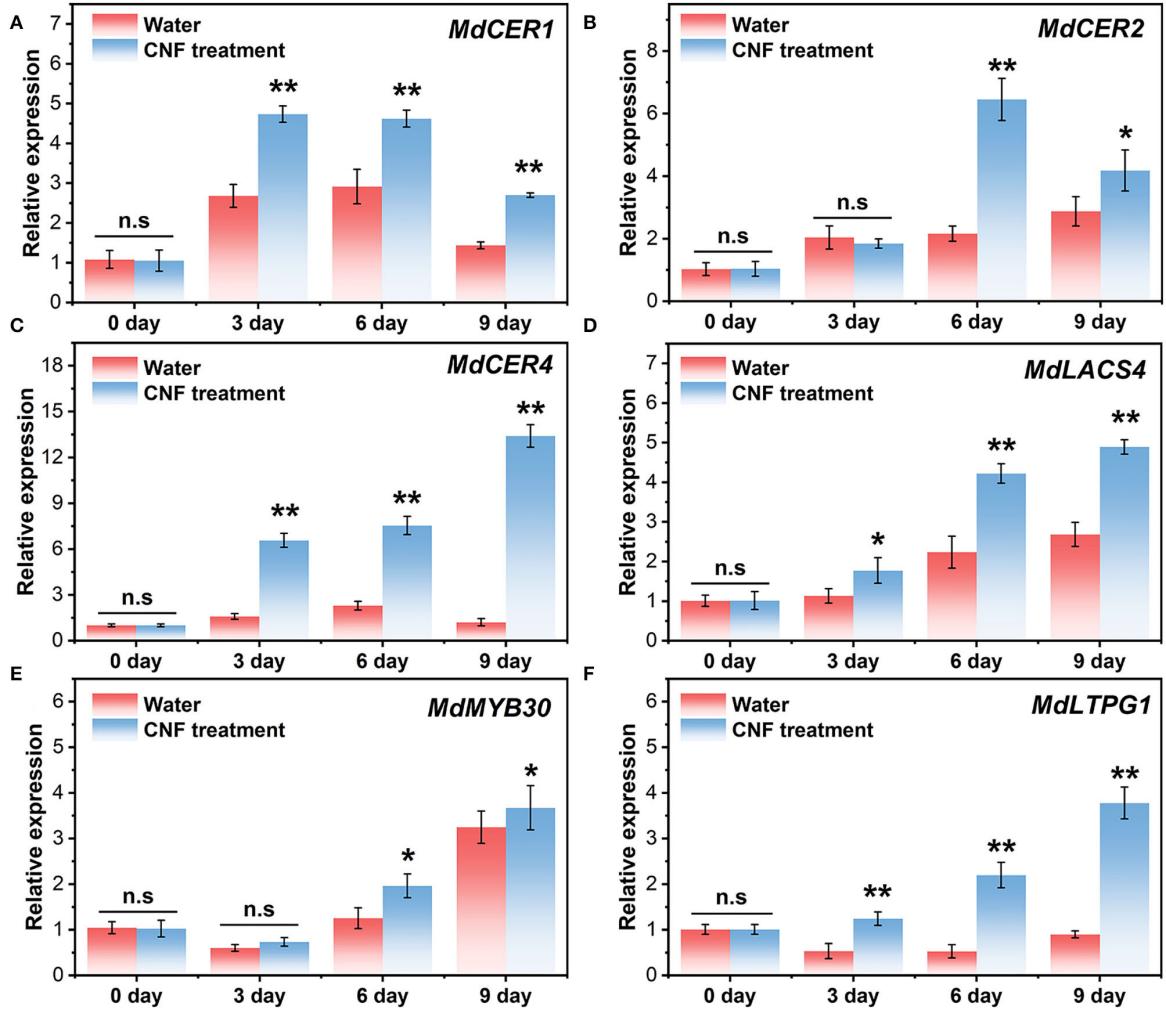
Relative expression of **(A)** *MdCER1*, **(B)** *MdCER2*, **(C)** *MdCER4*, **(D)** *MdLACS4*, **(E)** *MdMYB30*, and **(F)** *MdLTPG1* in the control and CNF-treated fruits. The asterisks indicate a statistically significant difference (two-tailed Student *t* test, *P < 0.05, **P < 0.01). n.s. represents no significant difference (*P* > 0.05). Error bars, mean ± standard deviation. Six biological replicates.

The phenotypic difference between the control samples and CNF-treated fruits might be relative to their gene expression. Figure 6 shows the relative expression of *MdCER1, MdCER2, MdCER4, MdLACS4, MdMYB30*, and *MdLTPG1* in the two groups. *MdCER1, MdCER2, MdCER4*, and *MdLACS4* belonged to structural genes involved in wax biosynthesis (53–55). These genes were upregulated during postharvest storage, suggesting that water loss stress could induce their expression. Intriguingly, the four genes in CNF-treated fruits had higher expression with more significant up-regulation tendency compared with the control groups (Figures 6A–D). Particularly, the *MdCER4* relative expression in the CNF-treated samples was more than 10 times higher than that in the water-treated fruits (Figure 6C). In *Arabidopsis thaliana, AtCER4* gene expression is associated with fatty alcohol biosynthesis (56) and is highly homologous to the MdCER4 protein in apples (57), which explains the sharp increase in the fatty alcohol content in the cuticular wax of fruits treated with CNF (Figures 4B–D). *MdMYB30* is a transcription factor that regulates cuticular wax biosynthesis at the transcriptional level and plays an important role in wax biosynthesis and disease resistance in apples (58). Figure 6E shows that the relative expression of *MdMYB30* in the two samples was slightly down-regulated in the beginning stage of storage (3 days) and then rapidly up-regulated, while CNF treatment induced the more relative expression after 6 days of storage. The function of *MdLTPG1* might be related to lipid export (57, 59). A slight downregulation of relative expression of *MdLTPG1* was observed in the control samples (Figure 6F). In contrast, the relative expression of *MdLTPG1* in CNF-treated fruits increased dramatically with the increase in storage (Figure 6F). In addition, the correlation analysis of wax component content and gene expression level between the two groups showed that *MdLTPG1* expression level was weakly correlated (0.3 < R^2^ < 0.5) with fatty alcohol content in the control group (Supplementary Figure S5A), while it was moderately correlated (0.5 < R^2^ < 0.8) with CNF treatment group (Supplementary Figure S5B). These results elucidated that the CNF preservative intensively induced the biosynthesis of epidermal wax, which corresponded to the analysis of apple fruit epidermal wax.

## Conclusions

In summary, we demonstrated an effective nanoscale fruit preservation agent. The preservative agent composed of CNFs directly derived from the natural plants, and thus it was renewable, degradable, and sustainable. The CNF preservative agent–treated apple fruits exhibited better appearance acceptability even after 10 days of storage. The epicuticular morphological observation and wax compound analysis indicated that the CNFs could improve the wax structure and components to achieve the fresh-keeping function of fruits. The analysis of biophysical and biochemical properties further demonstrated that the CNF preservative agent efficiently inhibited respiration rate, ethylene production, and cell membrane permeability to maintain the firmness and weight of the fruits during postharvest storage. The gene expression patterns also illustrated that the CNF preservative could intensively induce upregulation of the biosynthesis-related genes of epidermal wax. Therefore, this study provided an available renewable fruit preservative agent with nano-characteristics and high efficiency.

## Data Availability Statement

The original contributions presented in the study are included in the article/Supplementary Material, further inquiries can be directed to the corresponding authors.

## Author Contributions

YW designed the experiment and performed the study. XW, TZ, FZ, SZ, and CY analyzed the data. XW and KY wrote the paper. All authors discussed the results. All authors contributed to the article and approved the submitted version.

## Funding

The work was financially supported by National Key Research and Development Program of China (2018YFD1000200), Taishan Industrial Leading Talents (LJNY202026), China Agriculture Research System of MOF and MARA(CARS27), Natural Science Foundation of Shandong Province (ZR2020ZD43).

## Conflict of Interest

The authors declare that the research was conducted in the absence of any commercial or financial relationships that could be construed as a potential conflict of interest.

## Publisher's Note

All claims expressed in this article are solely those of the authors and do not necessarily represent those of their affiliated organizations, or those of the publisher, the editors and the reviewers. Any product that may be evaluated in this article, or claim that may be made by its manufacturer, is not guaranteed or endorsed by the publisher.
