# Supplementary material for: Cellulose Nanofibers Extracted From Natural Wood Improve the Postharvest Appearance Quality of Apples
Source: Front Nutr. 2022 May 13;9:881783. doi: 10.3389/fnut.2022.881783 (PMC9136226; doi:10.3389/fnut.2022.881783)
Supplement: Supplementary file 1 [file Data_Sheet_1.PDF]

Supporting Information

Cellulose nanofibers extracted from natural wood improve the appearance quality of fruit

Yongxu Wang[1,2], *Jing Zhang*[2], *Xinjie Wang*[2], *Tingting Zhang*[1,2], *Fujun Zhang*[1,2], *Shuai Zhang*[2], *Yuanyuan Li*[2], *Wensheng Gao*[3], *Chunxiang You*[2], *Xiaofei Wang*[2*], *Kun Yu*[1*]

1.Xinjiang Production and Construction Corps Key Laboratory of Special Fruits and Vegetables Cultivation Physiology and Germplasm Resources Utilization, Department of Horticulture, College of Agriculture, Shihezi University, Shihezi, 832003, Xinjiang, China.

2. National Key Laboratory of Crop Biology, MOA Key Laboratory of Horticultural Crop Biology and Germplasm Innovation, College of Horticulture Science and Engineering, Shandong Agricultural University, Tai'An 271018, Shandong, P. R. China.

3. Shandong Agricultural Technology Extension Center, Jinan, 250013, Shandong, China

*E-mail: yukun409@163.com (K.Y.)

*E-mail: xfwang2004@163.com (X.F.W.)

Supplementary Table 1 Primers for qRT-PCR

Numbers	Gene	Forward primer 5'-3'	Reverse primer 3'-5'
1	*MdActin*	ACACGGGGAGGTAGTGACAA	CCTCCAATGGATCCTCGTTA
2	*MdCER1*	GAAGACTCCCGCATCTCTTG	TCATGCTCCTTCCAACCTTC
3	*MdCER2*	ATCCGGATAACCGAAACGGG	AGGCAGAAAACGCATCTCCA
4	*MdCER4*	CTCTCAATACTTTGGGAGC	AGTTTTTCTTGGAGCAGCC
5	*MdLACS4*	AAGACCATGTTCAATCTTGCATATTC	GTATAAGCCGCACCCTACCAC
6	*MdMYB30*	TAATTACCTCAGGCCAGGGA	TTTTTAATGTCATTGTCAGTTCTC
7	*MdLTPG1*	AAATGATTGCGGTGTTGTT	TCCAGTAGTGGGAGTTGCT

Supplementary Table 2 The content of cuticular wax composition in apple during storage at room temperature (±25℃).

No.	Compounds	Code	Chemical formula	Retention time	Molecular weight	Content (μg cm^{-2})					
						0 day		ck 10day		CNF treatment 10day	
						Alkanes					
1	Heneicosane	C21	$C_{21}H_{44}$	18.631	296.57	N.D.	x	N.D.	x	0.2310 ± 0.1153	a
2	Tricosane	C23	$C_{23}H_{48}$	21.790	324.63	0.5998 ± 0.0682	b	N.D.	x	1.5035 ± 0.1341	a
3	Pentacosane	C25	$C_{25}H_{52}$	25.145	352.68	1.3666 ± 0.1141	a	0.2098 ± 0.0323	b	0.4526 ± 0.0323	b
4	Hexacosane	C26	$C_{26}H_{54}$	26.332	366.71	0.2696 ± 0.0816	a	0.2737 ± 0.0619	a	0.2210 ± 0.0524	a
5	Heptacosane	C27	$C_{27}H_{56}$	28.579	380.73	2.6159 ± 0.0743	b	2.7147 ± 0.4167	b	3.4329 ± 0.1488	a
6	Octacosane	C28	$C_{28}H_{58}$	30.195	394.76	2.3478 ± 0.2477	a	1.4620 ± 0.1603	b	2.5699 ± 0.2709	a
7	Nonacosane	C29	$C_{29}H_{60}$	32.122	408.79	131.6761 ± 18.2189	a	113.6274 ± 13.6409	b	137.7646 ± 11.7391	a
8	Triacontane	C30	$C_{30}H_{62}$	33.433	422.81	1.2219 ± 0.1405	a	0.9831 ± 0.2270	a	1.3598 ± 0.0928	a
9	Hentriacontane	C31	$C_{31}H_{64}$	34.996	436.84	1.7365 ± 0.2328	a	1.5262 ± 0.2766	a	1.4534 ± 0.4317	a
						Fatty alcohols					
10	Docosanol	C22	$C_{22}H_{46}O$	26.485	326.60	N.D.	x	N.D.	x	0.7818 ± 0.1025	a
11	Tetracosanol	C24	$C_{24}H_{50}O$	28.127	356.67	0.4863 ± 0.0549	b	0.7377 ± 0.2901	b	3.5529 ± 1.2096	a
12	Hexacosanol	C26	$C_{26}H_{54}O$	29.416	382.71	0.3065 ± 0.0645	b	8.5668 ± 0.7082	a	7.7399 ± 0.3566	a

13	Heptacosanol	C27	$C_{27}H_{56}O$	34.198	396.73	2.9101 ± 0.3227	**a**	0.2497 ± 0.0408	**b**	0.8272 ± 0.1022	**b**
14	Octacosanol	C28	$C_{28}H_{58}O$	34.883	410.76	6.9219 ± 0.6182	**b**	3.3898 ± 0.6830	**c**	13.5491 ± 4.2221	**a**
15	10-Nonacosanol	10-C29	$C_{29}H_{60}O$	35.260	424.78	78.6123 ± 3.9895	**b**	66.9925 ± 1.6245	**c**	126.9220 ± 12.3263	**a**
16	Triacontanol	C30	$C_{30}H_{62}O$	38.683	438.81	3.2146 ± 0.4370	**b**	1.9811 ± 0.4575	**b**	8.6040 ± 3.2352	**a**
								**Fatty aldhydes**			
17	Docosanal	C22	$C_{22}H_{44}O$	29.325	324.58	0.3407 ± 0.0348	**a**	0.4075 ± 0.0810	**a**	0.7064 ± 0.0948	**a**
18	Hexacosanal	C26	$C_{26}H_{52}O$	30.437	380.69	1.7614 ± 0.1570	**b**	1.7806 ± 0.2473	**b**	4.0055 ± 0.6274	**a**
19	Heptacosanal	C27	$C_{27}H_{54}O$	32.207	394.72	0.6772 ± 0.1148	**a**	N.D.	**x**	N.D.	**x**
20	Octacosanal	C28	$C_{28}H_{56}O$	33.737	408.74	2.7626 ± 0.1781	**c**	4.7837 ± 0.5644	**b**	9.5634 ± 1.1764	**a**
21	Nonacosanal	C29	$C_{29}H_{58}O$	35.303	422.77	0.9056 ± 0.0991	**b**	2.1319 ± 0.1321	**a**	2.4453 ± 0.4941	**a**
22	Triacontanal	C30	$C_{30}H_{60}O$	36.890	436.80	7.6966 ± 1.2647	**c**	14.1397 ± 2.8117	**b**	20.9229 ± 3.3603	**a**
								**Free fatty acids**			
23	Palmitic Acid	C16	$C_{16}H_{32}O_2$	17.756	256.42	4.0301 ± 0.7250	**b**	3.0013 ± 0.2266	**c**	6.4851 ± 1.4055	**a**
24	Oleic Acid	C18:1	$C_{18}H_{34}O_2$	27.850	282.46	7.6682 ± 0.4551	**a**	1.5841 ± 0.1574	**b**	7.9995 ± 1.7454	**a**
25	Linoleic acid	C18:2	$C_{18}H_{32}O_2$	27.783	280.45	14.4414 ± 2.2684	**a**	2.3737 ± 0.4302	**c**	5.2259 ± 1.4123	**b**
26	Stearic acid	C18	$C_{18}H_{36}O_2$	20.781	284.48	1.5450 ± 0.1323	**b**	0.6106 ± 0.1785	**c**	3.0966 ± 1.2339	**a**

27	Eicosanoic acid	C20	$C_{20}H_{40}O_2$	24.286	312.53	0.2209 ± 0.0412	**b**	0.1877 ± 0.0498	**b**	0.9132 ± 0.2372	**a**
28	Behenic acid	C22	$C_{22}H_{44}O_2$	26.950	340.58	0.3139 ± 0.0813	**b**	0.2860 ± 0.0964	**b**	4.3526 ± 0.5583	**a**
29	Lignoceric acid	C24	$C_{24}H_{48}O_2$	30.750	368.64	1.2882 ± 0.1201	**b**	1.5307 ± 0.3462	**b**	4.6623 ± 1.0563	**a**
30	Hexacosanoic acid	C26	$C_{26}H_{52}O_2$	35.475	396.69	1.3093 ± 0.0983	**a**	0.2298 ± 0.0919	**c**	0.7895 ± 0.1501	**b**
31	Octacosanoic acid	C28	$C_{28}H_{56}O_2$	36.994	424.74	0.6464 ± 0.0885	**b**	0.7831 ± 0.1191	**b**	2.2774 ± 0.1850	**a**
32	Triacontanoic acid	C30	$C_{30}H_{60}O_2$	40.031	452.80	1.3030 ± 0.1206	**b**	1.0212 ± 0.1068	**b**	3.8771 ± 0.4750	**a**
								**Ketones**			
33	10-Nonacosanone	10-C29	$C_{29}H_{58}O$	43.975	422.77	0.8017 ± 0.1372	**a**	0.7100 ± 0.1080	**a**	0.7143 ± 0.1404	**a**
34	2-Nonacosanone	2-C29	$C_{29}H_{58}O$	44.742	422.77	1.4267 ± 0.1022	**a**	1.5737 ± 0.1744	**a**	N.D.	**x**
								**Esters**			
35	Ethyl linoleate	Ethyl-C16:1	$C_{20}H_{38}O_2$	26.958	310.51	0.5078 ± 0.0721	**b**	0.9013 ± 0.1447	**b**	2.3351 ± 0.1886	**a**
36	Butyl palmitate	Butyl-C16:1	$C_{20}H_{40}O_2$	27.233	312.54	N.D.	**x**	0.9013 ± 0.1447	**a**	0.3858 ± 0.1238	**a**
37	Ethyl linoleate	Ethyl-C18:2	$C_{20}H_{36}O_2$	27.042	308.50	18.2715 ± 0.7794	**b**	15.2636 ± 0.7190	**c**	25.9495 ± 2.4118	**a**
38	Ethyl octadecanoate	Ethyl-C18:1	$C_{20}H_{40}O_2$	27.375	312.53	4.1406 ± 0.4639	**b**	4.7210 ± 0.2235	**b**	9.1386 ± 0.7519	**a**
39	Pentyl linoleate	Pentyl-C18:2	$C_{23}H_{42}O_2$	31.758	350.22	1.1791 ± 0.1763	**b**	0.4066 ± 0.0970	**c**	2.5277 ± 0.6667	**a**
40	Propyl linoleate	Propyl-C18:2	$C_{21}H_{38}O_2$	30.542	322.53	6.9713 ± 0.5033	**a**	2.4114 ± 0.2344	**b**	1.2762 ± 0.1740	**c**

41	Butyl oleate	Butyl-C18:1	$C_{22}H_{42}O_2$	30.608	338.57	7.8031 ± 0.4604	**b**	3.5775 ± 0.3636	**c**	12.2223 ± 0.3173	**a**
42	Ethyl docosanoate	Ethyl-C22:1	$C_{24}H_{48}O_2$	34.842	368.64	N.D.	**x**	0.9508 ± 0.1312	**b**	2.5572 ± 0.0849	**a**
								**Triterpenoids**			
43	Ursolic acid	Ursolic acid	$C_{30}H_{48}O_3$	43.283	456.68	21.9530 ± 0.3569	**a**	23.1942 ± 1.0963	**a**	22.4892 ± 0.7890	**a**
44	Oleanolic acid	Oleanolic acid	$C_{30}H_{48}O_3$	43.883	456.68	6.1548 ± 0.2786	**a**	7.4372 ± 0.8496	**a**	6.2161 ± 0.3287	**a**
45	α-Amyrin	α-Amyrin	$C_{30}H_{50}O$	38.981	426.72	1.6130 ± 0.1135	**a**	1.7238 ± 0.1317	**a**	1.9162 ± 0.1168	**a**
46	Lupoel	Lupoel	$C_{30}H_{50}O$	39.099	426.72	0.3550 ± 0.0968	**a**	0.3633 ± 0.0496	**a**	0.3757 ± 0.0899	**a**
								**Sesquiterpenes**			
47	β-Farnesene	β-Farnesene	$C_{15}H_{24}$	15.808	204.35	0.2125 ± 0.0216	**a**	0.2504 ± 0.0197	**a**	0.2210 ± 0.0224	**a**
48	α-Farnesene	α-Farnesene	$C_{15}H_{24}$	16.025	204.35	8.4671 ± 1.0490	**a**	0.6732 ± 0.0561	**c**	1.8068 ± 0.2989	**b**
49	(E, E)-Farnesol	(E, E)-Farnesol	$C_{15}H_{26}O$	20.500	222.37	1.7107 ± 0.2278	**a**	0.4056 ± 0.0744	**b**	0.4885 ± 0.0872	**b**

Note: Different letters represent significant difference according to the Duncan's new multiple range test ($P < 0.05$). x indicates that the composition is not detected. Error bars, mean ± SD, 6 biological replicates.

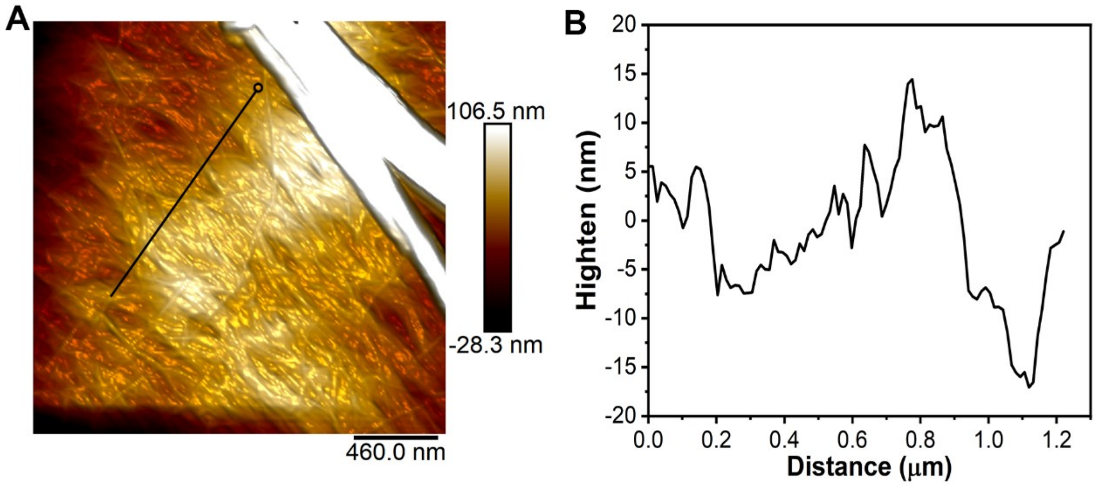

Supplemental Figure 1. (A) AFM image of CNF preservatives, and (B) corresponding height profile line.

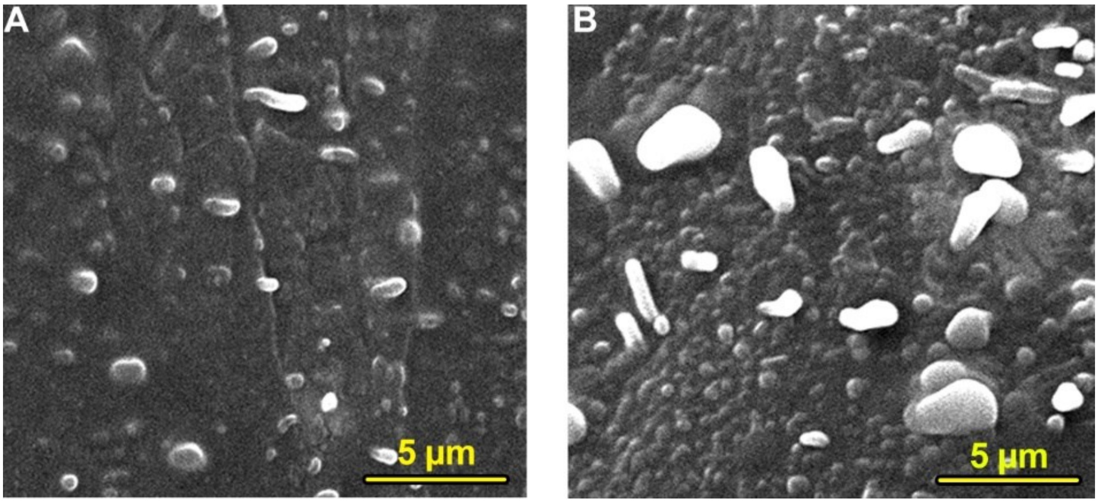

Supplemental Figure 2. Scanning electron microscopy observe the wax morphology of fruit epidermis after 10 days of control and CNF treatment. (A) Water. (B) CNF treatment.

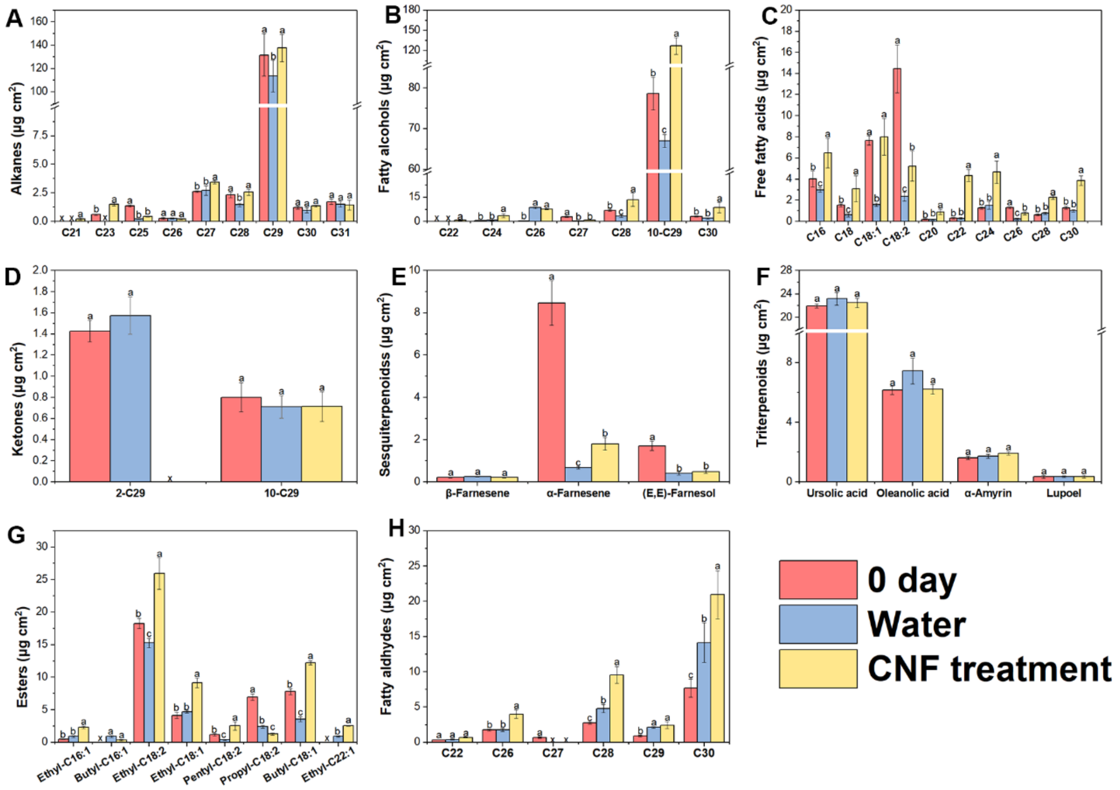

Supplemental Figure 3. Concentration of cuticular wax composition in the apples treated by CNF and water for 10 day. (A) Alkanes. (B) Fatty alcohols. (C) Free fatty acids. (D) Ketones. (E) Sesquiterpenoids. (F) Triterpenoids. (G) Esters. (H) Fatty aldehydes. Different letters represent significant difference according to the Duncan's new multiple range test ($P < 0.05$). x indicates that the composition is not detected. Error bars, mean ± SD, 6 biological replicates.

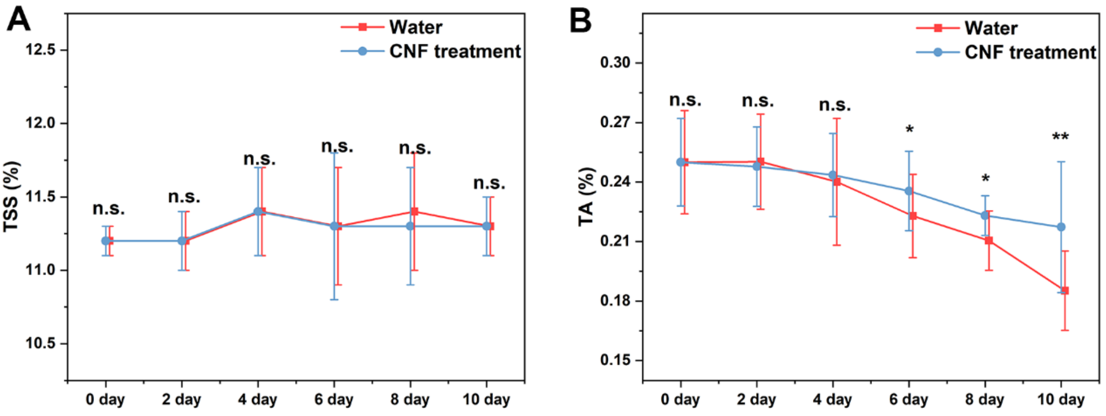

Supplemental Figure 4. (A) TSS content. (B) TA content. The asterisks indicate a statistically significant difference (two-tailed Student's t-test, *P < 0.05, **P < 0.01). n.s. represent no significant difference (P > 0.05). Error bars, mean ± SD. 6 biological replicates.

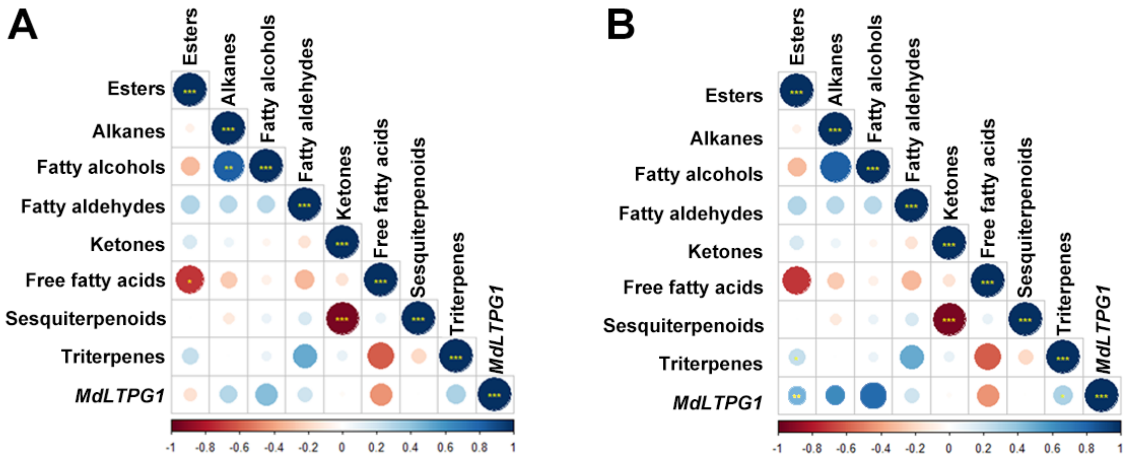

Supplemental Figure 5. Correlation analysis among wax chemical contents and MdLTPG1 expression level after the end of storage. (A) Control. (B) CNF treatment. The colored bars indicate the correlation coefficient (R^2) from low to high. The asterisks indicate a statistically significant difference ($^{*}P < 0.05$, $^{**}P < 0.01$, $^{***}P < 0.001$).
